# The Future of Prenatal Diagnosis and Screening

**DOI:** 10.3390/jcm3041291

**Published:** 2014-11-14

**Authors:** Eugene Pergament

**Affiliations:** Northwestern Reproductive Genetics, Inc., 142 East Ontario, Suite 525, Chicago, IL 60611, USA; E-Mail: e-pergament@northwestern.edu; Tel.: +1-312-888-9910

**Keywords:** embryonic development, genome analysis, gene therapy

## Abstract

The future of prenatal diagnosis and screening lies in developing clinical approaches and laboratory technologies applicable to genetic analyses and therapeutic interventions during embryonic development.

## 1. Introduction

In addressing the challenge of predicting the future of prenatal diagnosis and screening, the inimitable quotations attributed to Yogi Berra, New York baseball catcher (1951–1963) and manager (1963–1989), come immediately to mind. He is reportedly to have made the following three paradoxical statements: first, “It’s tough to make predictions, especially about the future”; followed by words of advice, “If you don’t know where you are going, you’ll end up someplace else”; and concluding with a somewhat cautionary note, “The future ain’t what it used to be”. 

The past half-century has been witness to the application of a series of clinical and laboratory technologies that have provided remarkable insights into the human genome. It is now possible to define the human genotype at distinct biological levels, chromosome, gene, nucleotide, as well as their intrinsic and extrinsic interactions culminating in phenotype. Nowhere have these technologies, and the genetic information generated from them, been more widely applied than in the case of prenatal testing, both screening and diagnosis. Each year hundreds of thousands of women and their partners now undergo genetic testing in the form of carrier screening for single gene mutations, prenatal screening for chromosome abnormalities, and/or diagnostic analysis following an invasive procedure (first trimester chorionic villus sample or midtrimester amniocentesis). The dynamism of the field of prenatal genetic diagnosis and screening is illustrated with the ongoing introduction of new technologies, such as non-invasive prenatal testing (NIPT), array comparative genome hybridization (aCGH), exome sequencing, whole genome amplification, and RNA sequencing. The time has come where testing of a pre-implantation embryo, post-implantation embryo or a fetus has the potential of revealing not simply individual, deleterious mutations but rather a series of genes and gene interactions that impact on different stages of life in both positive and negative manners. If, as anticipated, the technologies associated with single cell analysis of the transcriptome [1,2,3,4,5,6] and the expansion of the proteonome [7] become a clinical reality, the obvious question and past designation as to who and what are “normal” may no longer have any value or utility, losing meaning both on an individual basis as well as from a population perspective and just as dynamic has been the increasing sophistication in the prenatal analyses of the genome and its products, there has been virtually no change in parental choices following identification of any genetic mutation, which at present is either termination of pregnancy or continuation with birth of affected offspring. Incorporating all of this into a meaningful discussion on the future of prenatal diagnosis and screening leads to five premises: 

**Premise 1:** Whereas the field of reproductive genetics has achieved remarkable success in the diagnosis and screening of chromosome and single gene disorders, the goal of positive therapeutic interventions has yet to be achieved.

**Premise 2:** The etiology and pathogenesis of many genetic/developmental disorders are known and/or are being continuously refined and detailed.

**Premise 3:** There is a categorical imperative for the need to shift focus from late identification of genetic and developmental disorders of the fetus (*i.e.*, 9 weeks gestation and beyond) to the embryonic period (*i.e.*, pre-implantation to day 58 post-conception). 

**Premise 4:** Identification of genetic and developmental disorders in the embryonic period is insufficient; there is a need to develop therapeutic interventions that either correct, alleviate and/or minimize the phenotypic effects of any genetic and developmental disorder at the time of their potential genesis, namely, during embryogenesis.

**Premise 5:** Recognizing that there will be insufficient numbers of medical geneticists and genetic counselors to address the expansive genetic information generated by the multitude of technologies applicable to prenatal diagnosis and screening, the format of genetic counseling will be required to change in philosophy and in practice.

The following is a discussion elaborating on each premise.

**Premise 1:** Whereas the field of reproductive genetics has achieved remarkable success in the diagnosis and screening of chromosome and single gene disorders, the goal of positive therapeutic interventions has yet to be achieved.

The first premise is that, based on current approaches and practices, essentially there will be no change in the future of prenatal diagnosis and screening. Whereas over the past 50 years prenatal identification of genetic mutations and developmental malformations has become increasingly sophisticated and expansive, the choices following such identification, namely, pregnancy termination or the birth of an affected offspring, have in fact not changed. In opposition to pregnancy termination, it has been proposed that prenatal diagnosis and screening represent a failure of medicine in the classical sense. This failure of current practices of prenatal diagnosis and screening, it is claimed, was first articulated specifically in the Hippocratic Oath:

“I will prescribe regimens for the good of my patients according to my ability and my judgment and never do harm to anyone.”

“I will give no deadly medicine to any one if asked, nor suggest any such counsel; and similarly I will not give a woman a pessary to cause an abortion.” (Author emphasis.)

As part of the argument, it is evident that the distinction between a viable and nonviable fetus as a rationale and justification for termination of pregnancy is artificial on medical grounds alone, with no need for moral or religious considerations. Consequently, as presently conducted, it is argued, prenatal diagnosis and screening represents a violation of the basic tenet of medicine from the perspective of the fetus and ultimately is likely in many instances to be considered a failure on the part of the parents as well, especially those choosing pregnancy termination. The argument further suggests that the diagnosis of a genetic mutation and/or developmental anomaly made in the first trimester, *i.e.*, 9–11 weeks’ gestation, is also an acknowledgement of a medical failure, since from a developmental perspective, the damage, physical and/or functional, has already taken place many weeks earlier and as a result the best that can be expected from fetal therapy is partial but incomplete amelioration of the phenotypic effects of most genetic mutations and/or developmental anomalies.

**Premise 2:** The etiology and pathogenesis of many genetic/developmental disorders are known and/or are being continuously refined and detailed.

The human genome consists of coding and noncoding DNA sequences. Coded DNA comprise sequences that are transcribed into mRNA and eventually translated into proteins in the course of embryonic development. Whereas these coding sequences comprise less than 2% of the genome, they ultimately lead to the production of all human proteins, recognizing that other biological processes lead to the formation of many more unique proteins than the number of protein-coding genes [7]. In the case of prenatal genetic testing and evaluation, the current focus is identification of pathological conditions associated with clinically defined diseases and caused by sequence variation in genomic DNA. There are many different, clinically-significant forms of DNA sequence variation, ranging from polyploidy and aneuploidy to a change in a single nucleotide. The exact number of valid human protein-coding genes is unknown but estimates range from 2000–25,000; the average number of genes listed in human gene catalogs appears to be around 22,500 with uncertainty of the role of another 2000 genes, which appear to be retroposons, pseudogenes, or “orphan” DNA sequences, *i.e.*, non-human in origin [8]. In the case of pregnancy, either planned or in progress, current practices with regard to routine genetic testing can include the following, individually or combined: (1) carrier screening of prospective parent(s) of a defined set of disease-causing mutations based on population frequency, e.g., cystic fibrosis, spinal muscle atrophy, fragile X, as well as other, specific gene mutations based on family history; (2) visualization of fetal development by ultrasound, the earliest time usually no earlier than 9 weeks’ gestation; (3) first trimester screening for risk assessment of chromosome abnormalities, cardiac malformations and other developmental disorders; (4) non-invasive prenatal testing (NIPT) for aneuploidy involving chromosomes 21, 18, 21, X and Y and for microduplications/microdeletions associated with specific genetic syndromes, e.g., 22q deletions; (5) second trimester screening, particularly for open neural tube defects, as well as more detailed ultrasound evaluation at 18–20 weeks’ gestation; (6) diagnostic testing based on genetic analysis of chorionic villi or amniocytes, following invasive testing by first trimester chorionic villus sampling (CVS) or midtrimester amniocentesis, respectively; and, (7) in an increasing number of cases, microarray, exonic and whole genome analyses.

A number of Mendelian genes can serve as models wherein the complete spectrum of genetic and developmental effects of individual mutations has been defined. Examples would include, but not be limited to, sickle cell disease, Tay Sachs, spinal muscle atrophy, and cystic fibrosis. In the case of sickle cell disease (SCD), for example, every component of the pathogenesis has been defined including specific changes at the nucleotide level, the altered composition of the protein coded by the mutation(s), its effect on red cell morphology, the biochemical and physiological impact on somatic development, and the overall clinical expression as a direct consequence of the mutation(s). Patients with SCD experience multisystem complications comprising recurrent and painful vaso-occlusive crises, acute chest syndrome, splenic sequestration and pulmonary hypertension; organ damage begins at a young age and reduces life expectancy to half of the general population; SCD remains a highly morbid disease caused by chronic and progressive physical damage [9]. Despite knowledge of all facets of the disease process, the only available cure for SCD is hematopoietic stem cell transplant (HSCT), which has been demonstrated to improve overall survival by reversing organ damage and stabilizing CNS vasculopathy [10,11,12,13]. There is a series of medical as well as social barriers markedly prohibiting widespread usage of this approach, including lack of suitable HLA-matched donors, the risk of transplant-related toxicities, complications and even death, and the lack of information and limited understanding of HSCT by SCD family members [9]. Alternative methods and technologies such as population-wide pre-implantation genetic diagnosis and screening does not appear to be realistic or practical [14,15], whereas current forms of prenatal genetic diagnosis and screening with termination of affected pregnancies not only has moral, religious and legal constraints but also challenges the very core of medical philosophy and practice. 

**Premise 3:** There is a categorical imperative for the need to shift focus from late identification of genetic and developmental disorders of the fetus (9 weeks gestation and beyond) to the embryonic period (pre-implantation to day 58 post-conception).

It is generally understood that genetic disorders are developmental in nature and as such are formulated during embryogenesis; are already physically and/or functionally defined primarily before the beginning of the fetal stage; and, that developmental disturbances identified post-natally are in reality extensions of biological events occurring between three and eight weeks’ gestation. Consequently, the current practice of genetic testing following first trimester CVS or second trimester amniocentesis is applying prenatal diagnosis weeks far-too-late in pregnancy. Furthermore, as previously stated, when a genetic and/or developmental abnormality is identified using current technologies, the primary choices are usually limited to pregnancy termination or the birth of an affected offspring followed by ameliorative medical/surgical treatment. If this scenario is to be altered with a potentially positive outcome, alternative medical approaches and interventions that are not only realistic and practical but also acceptable to major sectors of any society, must be considered. Pre-implantation genetic diagnosis and screening is an obvious choice but it is proposed that in the future the focus of reproductive geneticists will be interventions during embryogenesis.

The initial impression is that pre-implantation evaluation, either of gametes (*i.e.*, polar bodies) or of embryos prior to transfer, easily meets the need of early identification of genetic and developmental disorders. There now is a relatively extensive worldwide body of knowledge and experience defining the field of pre-implantation genetic diagnosis and screening [14]. Over the past two decades thousands of couples have undergone pre-implantation diagnosis and screening for dozens of different genetic disorders following analysis either of polar body, day 3–4 embryo, or trophectoderm [14]. However, there are distinct disadvantages to this approach: pre-implantation genetic diagnosis and screening is costly, labor-intensive; limited to relatively few couples, not without controversy in regard to pre-implantation genetic screening [15] and, at present (2014) has a worldwide success rate of less than 25% in the “take-home-baby-rate” for each cycle of egg retrieval [14].

**Premise 4:** Identification of genetic and developmental disorders in the embryonic period is insufficient; there is a need to develop therapeutic interventions that will correct, alleviate or minimize the phenotypic effects of any genetic and developmental disorder at the time of their potential genesis, namely, during embryogenesis. 

### 1.1. A Historical Perspective

Prior to the introduction of prenatal diagnosis in the late 1960s, prospective parents at significant reproductive risk had limited preventive choices, either having no children by aborting each pregnancy, voluntary sterilization, sexual abstinence or by adoption. At the time of introduction of prenatal diagnosis by amniocentesis, the ultimate goal articulated by health professionals was fetal gene therapy, based in part in response to the controversy and rhetoric surrounding the morality and social implications of pregnancy termination based on genetic mutations. These debates have continued unabated to the present time, with one side viewing abortion following diagnostic testing for genetic disorders as a “logical or moral precedent for infanticide” [16] and their counterparts arguing that selective abortion prevents suffering of families and reduces the economic burdens to society [17]. Over the past two decades, there have been a series of publications focused on moral and ethical issues with respect to human fetal gene therapy [18,19,20,21,22,23]. Whereas the ethical and religious concerns about the current consequences of prenatal diagnosis and screening will likely remain unresolved, the future challenge to reproductive geneticists will be their creative ability in devising the technological means to: (1) physically and functionally define comprehensive embryonic development; and (2) be able to act on that information so as to modify specific forms of embryonic mal-development (e.g., open spinal defects) toward a positive outcome. There is likely to be a more general understanding and acceptance that with effective oversight, gene therapy during embryonic development is likely to be an acceptable medical goal, if it: (1) demonstrably improves the health and wellbeing of individuals who would otherwise experience irreversible physical and functional damage before birth, thereby potentially avoiding the need for alternative techniques such as gamete donation, embryo selection, abortion, adoption or non-parenthood; and (2) demonstrates that the risk of germline gene mutation through *in utero* gene transfer is minimal or nonexistent [17].

There is general consensus by health professionals that whereas elective abortion following any form of prenatal testing may be expeditious, in reality it still connotes an unsatisfactory medical response and outcome, as does the birth of a developmentally compromised newborn. The focus of the past 30 years has been on refining genetic analyses of the fetus, including the application of fluorescent *in situ* hybridization (FISH), chromosome banding, aCGH, next generation sequencing and NIPT, but all applied in distinguishing “normal” from “affected”. There have been projects aimed at integrating multiple approaches to identify the functional element encoded in the human genome, e.g., ENCODE [24], however, the focus being children and adults, its applicability to embryonic development is questionable, since it has been well established that the clinical effects of a genetic change detected in utero does not always match the postnatal phenotype. More recently, a data base correlating prenatal copy number variations (CNV) with postnatal outcome has been established, with a focus on the autistic spectrum disorder [25].

When genetic testing by amniocentesis was clinically introduced in the 1960s, it was generally recognized and agreed upon that this approach was not the ultimate goal of prenatal diagnosis. While the idea of fetal gene therapy was envisioned as a distant but attainable goal, it was understood that ultimately prenatal diagnostic testing had to be complemented by some form(s) of therapy, be it genetic, medical or surgical. Fetal gene therapy is inherently flawed as the potential detrimental phenotypic effects of any genetic mutation, be it at the chromosomal or molecular level, have already occurred in the embryonic period. Albeit with some minor success, in utero treatment has been applied to fetuses at risk for a number of genetic and congenital anomalies, including congenital diaphragmatic hernia, cystadenomatoid malformation of the lung and saccrococygeal teratoma, shunts for uropathies and thoracic fluids, pharmacological therapies for congenital adrenal hyperplasia and neural tube defect as well as stem cell treatment of severe combined immunodeficiency disorder [26,27,28,29,30]. The insights gained over the past decade with regard to stem cell biology as well as the ability to manipulate genetic sequences are two of an increasing number of technologies whereby there now are opportunities to alter the course of abnormal development during the critical time of embryogenesis, as summarized next. 

### 1.2. Future Perspectives

In the 30th Anniversary Issue of Prenatal Diagnosis published in 2010, three paradigm shifts in pre-implantation and prenatal genetics were projected to occur by 2020, each in turn enhancing one ultimate goal of medical science, namely, “personalized medicine”:

(1) Epigenomics would become a reality in which regulatory regions of all known genes in all major cell types and their disease variants would have been analyzed and their influences would be understood during prenatal and early postnatal development when epigenetic mechanisms undergo establishment and maturation;
(2) Prevention of chromosome abnormalities by defining the biological processes associated with gamete maturation including chromosome pairing, synapsis and segregation of homologs, thereby ensuring that the euploid state would be obtained at conception and maintained during early embryonic development;
(3) Treatment of mendelian, mitochondrial and multifactorial diseases in the pre-implantation and embryonic periods of development, which would involve cost-effective sequencing of the human genome at the single gene level, the development of databases cataloging submicroscopic copy number variants and their role in disease, a complete understanding of the pathogenesis of both single gene mutations and complex diseases, and knowledge derived from the epigenome, all of which have become a reality over the past decade [31].

Such related technologies would make possible implementation of targeted molecular interventions and the realization of the full potential of a personalized genome analysis in the prenatal period of human development, a consequence of the synergy of epigenetics, pharmacogenetics, and the ability to physically and chemically manipulate cells, tissues or organ systems [31]. The additional challenge of germline gene therapy, rationalized in part by anticipated success in somatic gene therapy, must also be considered [32]. Such a challenge will result in debates over the moral and ethical consequence of such actions, namely, the potential and realization of altering the genome of future generations, but more importantly, addressing the categorical imperative of medicine of doing no harm, *i.e.*, a call to re-examine germline gene therapy as a responsible way to address the consequences of somatic gene therapy [32].

Gene therapy is likely to be first applied in pre-implantation embryos, namely, in the case of mitochondrial disorders, which in turn immediately raises a recurring concern of simultaneously altering the germline. Both in the United Kingdom and United States, governmental approval has been requested for placing a fertilized oocyte at risk for a mitochondrial disorder into the cytoplasm with normal mitochondria, thus resulting in a conception with three genotypes, *i.e.*, maternal, paternal and mitochondrial. The potential of manipulating the genetic inheritance of future offspring by modification of spermatozoa, oocytes and embryos prior to implantation can be criticized as being “high tech eugenics” and experimentation on humans. This criticism is a recurring theme consistently sounded following the introduction of new prenatal technology, e.g., when amniocentesis, chorionic villus sampling, *in vitro* fertilization (IVF) and pre-implantation genetic diagnosis (PGD) were each introduced. In the case of mitochondrial disorders, the combined application of several different technologies provides an opportunity for a carrier parent to ensure an offspring free of the clinical devastation as a consequence of dysfunctional mitochondria.

While the pre-implantation substitution of defective mitochondria represents a heritable change by virtue of the interaction of these organelles with the recipient nuclear genome, this approach does not address nuclear genetic mutations, e.g., the over six thousand single gene mutations, the hundreds of different kinds of chromosome abnormalities, and/or any of the multifactorial traits. It is likely that any attempt at prenatal genetic therapy during embryogenesis will require different types of molecular technologies for different developmental disorder. Targeted gene therapy in metabolic disorders restricted to an organ system, e.g., the liver in phenylketonuria or in maple syrup urine disease, would appear to be an acceptable approach, implemented as early as possibly in gestation in order to minimize any potential negative impact, particularly the development and function of the central nervous system [33]. 

With the ability to edit DNA at the nucleotide level by virtue of such technologies as CRISPR (Clustered Regularly Interspaced Short Palindromic Repeats), the “future is now” in the case of prenatal genetic therapy may no longer be a paradoxical statement [34]. The technology uses a nuclease, CRISPR-associated (Cas9) that complexes with small RNAs as guides (gRNA) to cleave DNA in a sequence-specific manner upstream of the protospacer adjacent motif (PAM) in any genomic location. CRISPR editing technology has been successfully applied to model organisms, including nematodes, zebrafish, fruit flies and most recently in mammals, demonstrating its broad utility and specificity for editing genes regardless of their source and type [34]. It may be possible for the CRISPR technology to be applied to genes associated with genetically-determined forms of autism, addressing the question, “Could autism be treated prenatally?” [35,36]. For many genetic diseases expressed at birth and childhood, it is well recognized that their biological effects actually manifest during embryogenesis. Therefore, to be most effective, the future in prenatal diagnosis and screening should involve the development and application of technologies capable of identifying genetic disorders as early as possible in gestation, either in the pre-implantation or embryonic time periods, and then to apply corrective gene therapies that minimize, if not completely eliminate, their negative phenotypic impact on somatic development and/or intellectual functions. This will be an especially arduous scientific journey, requiring novel technologies involving embryonic visualization [37], gene manipulations [33] and/or transplantation of genetically-altered mesenchymal stem cells [27], all with the goal to ameliorate potential physical and functional damage. It is anticipated that the first effective therapies would be applied to a collective group of monogenic disorders for the purpose of achieving phenotypic cure early in the disease process and before the development of permanent organ damage [29].

One consequence of the development and application of individualized gene therapies will likely be challenges to the relative autonomy of parental decision-making. In fact, in societies practicing a public health model, challenges to parental autonomy concerning various aspects of genetic testing have already been accepted. In the case of private health care models, e.g., the United States, individual choice concerning various forms of genetic testing is often considerably modulated by the professional societies, which, in turn, influence another important factor in parental decision-making, namely, insurance coverage. Therefore, although the basic right of reproductive choice, of when, with whom, and how to reproduce, continues to be articulated by Western societies, parental autonomy has been, and will continue to be, moderated by health care providers, particularly as new technologies are applied in prenatal diagnosis. What has been increasingly contentious in Western societies, primarily on ethical and moral grounds, is the granting of broad autonomy to prospective parents when the choice includes abortion. Over the coming decades, it will be the medical consequences and available choices following prenatal diagnosis that will be expanded to include pre-implantation and embryonic gene therapies. And, if practiced in the pre-implantation embryo, it must be understood and accepted that any form of gene therapy will likely involve altering the germ line as well as the soma. 

## 2. Conclusions

How in the foreseeable future is genomic medicine to influence pregnancy management and pregnancy outcome? New genetic technologies as well as new therapeutic strategies must be developed if embryonic care is to include expanded choices of action beyond current means [2,3,4,5,6,7]. In other words, there must be a synergy of the ability to define *in vivo* embryonic development at all biological levels, *i.e.*, cell, tissue and organ [38], along with the technical means to repair, alter or at least minimize the phenotypic effects of potentially negative mutations [39,40,41,42], Thus, there should be a rapid and energetic shift in the research focus of prenatal diagnosis and screening, and a call for an expansion of all future prenatal studies to include therapeutic strategies other than current choices. This requires that the embryo must be completely defined not only at the genomic level but also analyzed at all stages of embryonic development the transcriptome, its functional pathways, as well as its intrinsic and extrinsic interactions [43,44]. This challenge is heightened by the knowledge that the oocyte [45], spermatozoa [46] and fertilized oocyte are characterized by high rates of spontaneous mutations, thereby diminishing the impact of familial inheritance [47]. Until new therapeutic strategies are developed and incorporated into the management of embryonic development, only then will a more complete and personalized form of medicine be attained.
